# Relaxation-Compensated Chemical Exchange Saturation Transfer MRI in the Brain at 7T: Application in Relapsing-Remitting Multiple Sclerosis

**DOI:** 10.3389/fneur.2022.764690

**Published:** 2022-02-25

**Authors:** Kristin P. O'Grady, Sanjana Satish, Quinn R. Owen, Bailey A. Box, Francesca Bagnato, Anna J. E. Combes, Sarah R. Cook, Holly James Westervelt, Haley R. Feiler, Richard D. Lawless, Asha Sarma, Shekinah D. Malone, Josephine M. Ndolo, Keejin Yoon, Richard D. Dortch, Baxter P. Rogers, Seth A. Smith

**Affiliations:** ^1^Vanderbilt University Institute of Imaging Science, Vanderbilt University Medical Center, Nashville, TN, United States; ^2^Department of Radiology and Radiological Sciences, Vanderbilt University Medical Center, Nashville, TN, United States; ^3^Neuroimaging Unit, Division of Neuroimmunology, Department of Neurology, Vanderbilt University Medical Center, Nashville, TN, United States; ^4^Department of Neurology, Nashville VA Medical Center, TN Valley Healthcare System, Nashville, TN, United States; ^5^Division of Behavioral and Cognitive Neurology, Department of Neurology, Vanderbilt University Medical Center, Nashville, TN, United States; ^6^School of Medicine, Meharry Medical College, Nashville, TN, United States; ^7^Department of Translational Neuroscience, Barrow Neurological Institute, Phoenix, AZ, United States; ^8^Department of Psychiatry and Behavioral Sciences, Vanderbilt University Medical Center, Nashville, TN, United States; ^9^Biomedical Engineering, Vanderbilt University, Nashville, TN, United States

**Keywords:** chemical exchange saturation transfer (CEST), glutamate, multiple sclerosis, metabolic imaging, ultrahigh field, apparent exchange-dependent relaxation (AREX), cognition

## Abstract

Chemical exchange saturation transfer (CEST) magnetic resonance imaging (MRI) can probe tissue biochemistry *in vivo* with high resolution and sensitivity without requiring exogenous contrast agents. Applying CEST MRI at ultrahigh field provides advantages of increasing spectral resolution and improving sensitivity to metabolites with faster proton exchange rates such as glutamate, a critical neurotransmitter in the brain. Prior magnetic resonance spectroscopy and CEST MRI studies have revealed altered regulation of glutamate in patients with multiple sclerosis (MS). While CEST imaging facilitates new strategies for investigating the pathology underlying this complex and heterogeneous neurological disease, CEST signals are contaminated or diluted by concurrent effects (e.g., semi-solid magnetization transfer (MT) and direct water saturation) and are scaled by the T_1_ relaxation time of the free water pool which may also be altered in the context of disease. In this study of 20 relapsing-remitting MS patients and age- and sex-matched healthy volunteers, glutamate-weighted CEST data were acquired at 7.0 T. A Lorentzian fitting procedure was used to remove the asymmetric MT contribution from CEST z-spectra, and the apparent exchange-dependent relaxation (AREX) correction was applied using an R_1_ map derived from an inversion recovery sequence to further isolate glutamate-weighted CEST signals from concurrent effects. Associations between AREX and cognitive function were examined using the Minimal Assessment of Cognitive Function in MS battery. After isolating CEST effects from MT, direct water saturation, and T_1_ effects, glutamate-weighted AREX contrast remained higher in gray matter than in white matter, though the difference between these tissues decreased. Glutamate-weighted AREX in normal-appearing gray and white matter in MS patients did not differ from healthy gray and white matter but was significantly elevated in white matter lesions. AREX in some cortical regions and in white matter lesions correlated with disability and measures of cognitive function in MS patients. However, further studies with larger sample sizes are needed to confirm these relationships due to potential confounding effects. The application of MT and AREX corrections in this study demonstrates the importance of isolating CEST signals for more specific characterization of the contribution of metabolic changes to tissue pathology and symptoms in MS.

## Introduction

Cognitive impairment is a significant symptom of relapsing-remitting multiple sclerosis (RRMS), affecting 40–70% of patients and occurring early in the disease course. Affected domains include information processing speed, memory, verbal fluency and executive function (1). Despite a significant impact on quality of life, the pathological substrates of cognitive impairment are poorly understood. Understanding its underlying mechanisms would help inform therapeutic decisions and advance development of adequate interventions and therapies.

While lesion load and location in cortical and deep gray matter (GM) and in white matter (WM) tracts have all shown associations with cognitive status, specific deficits, and cognitive deterioration over time (2), fully understanding the complex pathophysiology of MS requires imaging methods more specific to metabolic changes. Magnetic resonance imaging (MRI) methods that can probe brain pathophysiology and metabolism *in vivo* are needed to elucidate the mechanisms that underlie cognitive impairment.

Glutamate is the main excitatory neurotransmitter in the brain; dysregulation of glutamate homeostasis is implicated in various neuropsychiatric disorders. In MS, glutamate excitotoxicity, i.e., axonal damage and neuronal death caused by increased extracellular glutamate levels, has been implicated as a potential mechanism linking inflammation and neurodegeneration (3, 4). Previous investigations have used *in vivo* proton MR spectroscopy (1H-MRS) to probe several metabolites, including glutamate, in MS lesions and normal-appearing white matter (NAWM) (5). Glutamate was found to be elevated in acute WM lesions and NAWM compared to healthy control tissue (6), and an increased glutamate/*N*-acetylaspartate ratio in NAWM was predictive of longitudinal declines in brain volume, MS Functional Composite scores, and Paced Auditory Serial Addition Test (PASAT) scores (a commonly-used test of processing speed) at 5-year follow-up (7). In contrast, lower glutamate levels measured with MRS in GM regions have been correlated with worse visuospatial memory (8).

Chemical exchange saturation transfer (CEST) MRI provides another means to perform molecular imaging and detect endogenous, mobile biomolecules such as proteins, peptides, and metabolites (e.g., glutamate, creatine, myo-inositol) with high sensitivity. In CEST imaging, the water-exchangeable protons of solutes (e.g., amide, hydroxyl, amine, or imino protons) are labeled via saturation with frequency-selective, narrow-bandwidth RF irradiation. Forward and back chemical exchange between protons in the irradiated solute pool and protons in the bulk water pool are constantly occurring. Through repeated saturation and exchange, saturated protons accumulate in the water pool and decrease the water signal, which can be detected (9–11). Physiologically relevant, millimolar concentrations of endogenous molecules can be detected with CEST MRI given sufficient saturation and chemical exchange rate (12). Ultrahigh field MRI (7.0 Tesla and higher) is advantageous for CEST imaging because the spectral resolution and sensitivity to faster proton exchange rates are improved, and a longer water T_1_ increases the CEST effect (10). These features are important to glutamate-weighted CEST (“GluCEST”) (13–16) due to the intermediate/fast exchange rate of glutamate amine protons and resonance frequency (Δω = 3.0 ppm) closer to that of water relative to other common CEST targets (e.g., slow-exchanging amide protons in proteins at Δω = 3.5 ppm).

CEST MRI has been applied in many preclinical and clinical studies of diseases, including MS (16–21), to probe changes in tissue biochemistry with greater coverage and resolution relative to other metabolic imaging techniques such as MR spectroscopy. Nevertheless, it is important to note that several factors can confound or dilute the CEST contrast attributed to a molecule of interest. CEST is sensitive to the choice of saturation parameters (saturation pulse power and duration), field strength, direct water saturation (spillover effect), and T_1_ relaxation time of the free water pool. The semisolid macromolecular components in tissues participate in magnetization transfer (MT) which imparts a broad, asymmetric baseline convolved on the measured CEST spectrum (12, 22). Of note, the MT asymmetry is substantially larger than the CEST effect, which can diminish the detection of small changes in the CEST spectrum. Conventional approaches to quantifying CEST data involve computing the magnetization transfer ratio (MTR) asymmetry which compares the signal at the frequency of interest (Δω) to that at a reference frequency (typically -Δω) after correction for B_0_ inhomogeneities. This analysis does not account for the broad, asymmetric baseline in the CEST spectrum and does not isolate the CEST signal from other tissue changes that may occur simultaneously in disease [e.g., demyelination, water content changes due to inflammation and edema (23, 24)]. Several methods to isolate CEST contrast for a target molecule from the confounding spillover, MT, and T_1_ effects have been developed, including modeling with modified Bloch-McConnell equations (25), fitting the CEST spectrum with multiple Lorentzian line shapes (26, 27), and inverse z-spectrum analysis approaches (28). The apparent exchange-dependent relaxation (AREX) correction for CEST quantification is one such correction that has been shown to reduce the influence of these competing effects (28–31).

Our prior study of glutamate-weighted CEST MRI in MS using a conventional asymmetry analysis showed increased GluCEST contrast in the prefrontal cortex of patients with MS relative to controls, and GluCEST in cortical regions correlated negatively with measures of cognitive function (16). In the current study, we sought to examine glutamate-weighted CEST at 7.0 T in patients with MS with two additional measures taken to isolate the CEST signal from confounding effects. First, we used a Lorentzian fitting procedure to remove the asymmetric MT contribution from CEST z-spectra. We then applied the AREX correction to reduce the influences of spillover, MT, and T_1_ effects on CEST signal quantification. MT-corrected AREX contrast in NAWM and normal-appearing GM (NAGM) and in MS lesions was compared to healthy tissue, and associations with clinical measures of disability and cognitive impairment were examined.

## Materials and Methods

### Study Participants

The studies involving human participants were reviewed and approved by the Vanderbilt University Institutional Review Board, and the participants provided their written informed consent prior to examination. Twenty patients with a diagnosis of relapsing-remitting MS according to the revised 2010 McDonald Criteria (32) (19–56 years old, mean age 38.5 ± 10.6 years, 12F/8M, Expanded Disability Status Scale (EDSS) score range of 0–4.5 and median of 1.5 determined by clinical examination) and 20 age- and sex-matched healthy volunteers (23–57 years old, mean age 39.2 ± 10.8 years, 13F/7M) were enrolled. All participants underwent a brain MRI at 7 Tesla (7.0 T) field strength, and a subset of the participants completed a neuropsychological assessment using the Minimal Assessment of Cognitive Function in MS (MACFIMS) battery (33) and two additional tests of processing speed and motor speed [Simple Reaction Time with one stimulus and Choice Reaction Time with four stimuli (34)]. Only the 2 second interval version of the PASAT was administered. Nineteen healthy volunteers and 15 MS patients completed the neuropsychological battery, and the remaining 5 patients completed a subset of tests. Complete demographic data are shown in Table 1. The Word Reading score from the Wide Range Achievement Test, 4^th^ edition (WRAT4), was used as an estimate of premorbid ability and age-corrected, standard scores did not differ between groups.

**Table 1:** Demographic information.

	**MS patients (*n* = 20)**	**Healthy volunteers (*n* = 20)**
Females	12 (60%)	13 (65%)
Age, mean ± SD	38.5 ± 10.6 years	39.2 ± 10.8 years
Years of education, mean ± SD	15.4 ± 2.9 years^*^	17.2 ± 2.3 years
WRAT4 word reading age-corrected standard score, mean ± SD	108.5 ± 7.5	112.1 ± 9.3
EDSS, median (range)	1.5 (0–4.5)	-
Disease duration, mean ± SD (range)	8.8 ± 7.9 years (0.5–29)	-

*MS, multiple sclerosis; EDSS, Expanded Disability Status Scale*.

*WRAT4 the Wide Range Achievement Test, 4^th^ edition, used as an estimate of premorbid ability*.

* *Patients with MS differ significantly from healthy volunteers (p < 0.05)*.

### MR Imaging

Brain MRI was performed using a Philips Achieva 7.0T MR Scanner (Philips Healthcare, The Netherlands) with a two-channel volume transmit, 32-channel receive head coil (Nova Medical, Wilmington, MA). The scan protocol included two whole brain anatomical scans for tissue segmentation. A three-dimensional (3D) magnetization prepared 2 rapid acquisition gradient echoes (MP2RAGE) (35) was acquired with a 3D Turbo Field Echo (TFE) sequence, 0.8 mm isotropic resolution, MP2RAGE_TR_ = 8.25 s, TR = 6.0 ms, TI_1_ = 1 s, TI_2_ = 3.3 s, TE = 2.6 ms, SENSE factor = 2 AP, 2 RL, FA_1_ = FA_2_ = 4°, and 9 min:04 s duration. A 3D magnetization prepared fluid-attenuated inversion recovery (MP-FLAIR) (36) was acquired with a 3D Turbo Spin Echo (TSE) sequence, 0.8 mm isotropic resolution, TR = 8 s, TI = 2425 ms, TE = 278 ms, SENSE factor = 2 AP, 2 RL, FA = 70°, and 9 min:36 s duration.

For glutamate-weighted CEST MRI, a 2D multi-shot TFE sequence was applied in a transverse orientation parallel to the anterior commissure - posterior commissure line with 1.5 x 1.5 x 10 mm^3^ resolution, TR = 4.1 ms, TE = 2.7 ms, SENSE factor = 2 (RL), FA = 10°, number of signal averages = 2, and 22 min:12 s duration. CEST data were acquired using a 4.25 μT (peak amplitude) pulse train of ten 60 ms Gaussian RF pulses (90% duty cycle, B_1rms_ = 1.97 μT over 670 ms pulse train) at 43 frequency offsets sampled asymmetrically between +/−5.0 ppm (−5.0, −4.6, −4.3, −4.0, −3.6, −3.3, −3.0, −2.6, −2.3, −2.0, −1.6, −1.3, −1.0, −0.8, −0.6, −0.4, −0.2, 0.0, 0.2, 0.4, 0.6, 0.8, 1.0, 1.2, 1.4, 1.6, 1.8, 2.0, 2.2, 2.4, 2.6, 2.8, 3.0, 3.2, 3.4, 3.6, 3.8, 4.0, 4.2, 4.4, 4.6, 4.8, and 5.0 ppm) with 13 interspersed, non-saturated reference images (S_0_) to correct for signal drift. A B_1_ map (dual-TR actual flip angle method, TR_1_/TR_2_/TE/FA = 35 ms/160 ms/2.0 ms/60°) (37) and a Water Saturation Shift Referencing (WASSR) sequence (38) were acquired for correction of B_1_ and B_0_ field inhomogeneities, respectively. For the WASSR sequence, a 0.5 μT, 100 ms Gaussian-shaped RF saturation pulse was applied at frequency offsets between +/−1.5 ppm with a step size of 0.1 ppm from +/−1.5 to +/−1.0 ppm and 0.05 ppm between +/−1.0 ppm with the following parameters: 2 x 2 x 10 mm^3^ resolution, TR = 5.6 ms, TE = 2.7 ms, FA = 10°, number of signal averages = 1, and 2 min:54 s duration. An R_1_ map for relaxation rate correction of CEST data was derived from an inversion recovery sequence acquired with the following parameters: a selective inversion recovery with a 3D multi-shot TFE readout, 5 slices, 1.1 x 1.1 x 2 mm^3^ resolution, TR = 4.1 ms, TE = 2.1 ms, SENSE factor = 2 (RL), FA = 15°, 14 TI values (6, 10, 16, 26, 42, 68, 110, 178, 288, 468, 760, 1233, 2000, and 8000 ms), pre-delay time of 2500 ms, composite inversion pulse duration = 6.5 ms, and 7 min total duration (39–41). The B_1_, WASSR, and inversion recovery sequences were acquired in the same geometry as the CEST slice.

### CEST Image Processing and Effect Isolation

Each CEST dynamic was registered to the first CEST dynamic using affine registration [FSL FLIRT (42, 43)] and normalized to a spline interpolation of the unsaturated S_0_ data to correct for signal drift (44) and generate the CEST z-spectrum (***Z***) for each voxel:


(1) $$\begin{array}{c} \text{Z}\left(\Delta \omega \right)=\frac{\text{S}\left(\Delta \omega \right)}{{\text{S}}_{0}\left(\Delta \omega \right)} \end{array}$$

A B_0_ frequency shift map was computed from the WASSR data and used to center the CEST z-spectra at Δω = 0 ppm on a voxel-wise basis (38). In prior studies, glutamate-weighted CEST (***GluCEST***) contrast was computed according to the following equation:


(2) $$\begin{array}{c} \text{GluCEST}=\frac{\text{S}\left(-\Delta \omega \right)-\text{S}\left(+\Delta \omega \right)}{\text{S}\left(-\Delta \omega \right)}\;\times \;100 \end{array}$$

where Δω is 3.0 ppm for sensitivity to glutamate amine protons (13, 16, 45). However, this CEST asymmetry equation does not completely remove the asymmetric MT effects from semi-solid macromolecules (e.g., myelin) which are known to be altered in MS pathology (23, 46–48). The AREX approach to quantifying CEST contrast mitigates some of the contributions of semi-solid MT effects, direct water saturation, and T_1_ weighting through an inverse subtraction analysis and correction for T_1_ effects (28–31), and recent studies aiming to improve the specificity of GluCEST have also incorporated a Lorentzian fitting procedure to remove the asymmetric MT baseline from the CEST spectra (14, 45). Here, we combined MT baseline removal and AREX quantification to probe the CEST effects at 3.0 ppm using the following procedure. First, a two-pool Lorentzian model consisting of MT and direct saturation (DS) pools was fit to the B_0_-corrected z-spectra on a voxel-wise basis. The labeled z-spectrum saturation (***Z***_***lab***_) can be represented as a baseline saturation (***Z***_***base***_, which would be 1 with perfect saturation efficiency) minus the Lorentzian components (***L***_***i***_):


(3) $$\begin{array}{c} {\text{Z}}_{\text{lab}}\;=\;{\text{Z}}_{\text{base}}\;-\;\sum_{i=1}^{\text{n}}{\text{L}}_{\text{i}}\left(\Delta \omega \right) \end{array}$$

where the Lorentzian line shape is represented by (49):


(4) $$\begin{array}{c} L_{i}\left(\text{Δω}\right)=100-\left(\frac{A_{n}}{1+4{\left(\frac{\text{Δω}-{\text{Δω}}_{\text{n}}}{{\text{σ}}_{n}}\right)}^{2}}\right) \end{array}$$

For each Lorentzian line shape included in the model, there are three unknown parameters: amplitude (***A**_**n**_*), width (**σ**_***n***_), and chemical shift (Δω_***n***_**)** of the *nth* pool, and Δω is the frequency of the off-resonance pulse. We opted to set fixed values for the chemical shift of each species, as these resonance frequencies are well established by previous research (50, 51). Chemical shifts of each species were set as: DS = 0 ppm and MT = −2.4 ppm. Table 2 contains the starting points and boundaries of the fit of the amplitude and width parameters. Initial values for simulations were based on Singh et al. (52). Z-spectral fitting was performed using the non-linear fitting “lsqnonlin” built-in MATLAB function. Once the fitting algorithm was complete, the MT contribution was removed from the measured CEST spectrum (***Z***) by subtracting the MT spectrum (the Lorentzian line shape from Equation 4 for the MT pool, which we have termed ***Z***_***LorentzMT***_) for each voxel, creating the corrected, normalized z-spectrum (***Z***_***corr***_):

**Table 2:** Initial estimates and bounds for Lorentzian fit of CEST data.

	**Amplitudes (%)**		**Width (ppm)**	
	**DS**	**MT**	**DS**	**MT**
Lower bound	20	0	0.1	10
Upper bound	100	90	5	100
Initial guess	60	35	2.55	55


(5) $$\begin{array}{c} {\text{Z}}_{\text{corr}}\left(\Delta \omega \right)\;=\;\text{Z}\left(\Delta \omega \right)-{\text{Z}}_{\text{LorentzMT}}\left(\Delta \omega \right) \end{array}$$

From the MT-corrected z-spectra, we calculated the corrected MTR asymmetry conventionally used to quantify CEST effects (30):


(6) $$\begin{array}{c} {\text{MTRasym}}_{\text{corr}}\;=\;{\text{Z}}_{\text{corr}}\left(-\Delta \omega \right)-{\text{Z}}_{\text{corr}}\left(+\Delta \omega \right) \end{array}$$

To remove MT, DS, and T_1_ effects and provide a more exchange-specific quantification of the CEST signal, we performed an inverse subtraction analysis and calculated the MT-corrected inverse z-spectrum (Equation 7), MT- and spillover-corrected inverse difference (Equation 8, ***MTR***_***RexCorr***_), and T_1_-corrected AREX contrast (Equation 9, ***AREX***_***corr***_) as described in prior studies (14, 28, 30) using the following equations:


(7) $$\begin{array}{c} \frac{1}{Z_{corr}}=\frac{1}{Z(\Delta \omega )}-\frac{1}{Z_{LorentzMT}(\Delta \omega )} \end{array}$$

(8) $$\begin{array}{c} MTR_{RexCorr}=\frac{1}{Z_{corr}(+\text{Δω})}-\frac{1}{Z_{corr}(-\text{Δω})} \end{array}$$

(9) $$\begin{array}{l} AREX_{corr}=\left(\frac{1}{Z_{corr}\left(+\Delta \omega \right)}-\frac{1}{Z_{corr}\left(-\Delta \omega \right)}\right)/T_{1}\\ \;=MTR_{RexCorr}\times R_{1} \end{array}$$

where Δω is 3.0 ppm for sensitivity to glutamate amine protons as before (13, 14, 16, 45). The R_1_ (longitudinal relaxation rate of the free water pool) values used in the AREX equation were derived from fitting the inversion recovery data as described previously (39–41). The five 2 mm-thick R_1_ map slices were averaged and registered to the 10 mm-thick CEST slice with affine registration using the 16^th^ CEST dynamic at Δω = −0.4 ppm as the target image. Registration results were checked in all cases using the target image (CEST dynamic at −0.4 ppm) as well as images at other offset frequencies (e.g., −3.0 ppm).

### Tissue Segmentation

T_1_-weighted MP2RAGE images were calculated from the complex image volumes acquired at TI_1_ and TI_2_ using the robust processing method described by O'Brien et al. (53). GM and WM tissue maps were segmented from the 3D MP2RAGE image using the “segment” tool in SPM12. Default settings were modified to improve performance on the 7.0 T MP2RAGE images which are inherently corrected for field inhomogeneities (bias regularization = “no regularization,” bias FWHM = “no correction,” and clean up procedure = “thorough”). Multi-atlas labeling using the spatially localized atlas network tiles (SLANT) method (54, 55) was applied to the MP2RAGE image to further divide the GM into distinct cortical regions (prefrontal, parietal, motor, somatosensory, temporal, and occipital cortices). MS lesions in the cortical GM were segmented manually from MP2RAGE images, and WM lesions were segmented manually using both MP2RAGE and MP-FLAIR images. The anatomical image volumes and associated tissue and lesion masks were registered to the inversion recovery image volume (TI = 1233 ms image as target) acquired in the same geometry as the CEST slice using the “Coregister: Estimate and Re-slice” tool with the “Normalized Mutual Information” function and nearest-neighbor interpolation in SPM12. Prior to registering these tissue and lesion masks (5 2 mm-thick slices) to the CEST slice (1 10 mm-thick slice), masks representing the 10 mm-thick slice were generated as follows: the GM, WM, and WM lesion masks retained voxels that occurred in at least 2 of the 5 slices, and the cortical lesion mask retained all lesion voxels occurring in any of the 5 slices due to the small size of these lesions. Lesion mask voxels were excluded from normal-appearing GM and WM masks. These 2D masks were then registered to the 2D CEST slice using the same affine transformation that registered the calculated 2D R_1_ map to the CEST slice (as described in Section CEST Image Processing and Effect Isolation above).

### CEST Analysis

MTR asymmetry, MTR_Rex_, and AREX maps were generated with and without the subtraction of the MT baseline component to visualize the influences of the asymmetric MT effect, direct saturation, and T_1_ on conventional CEST quantification procedures. Histograms of these contrasts calculated at Δω = 3.0 ppm were generated for each tissue region of interest (GM, WM, cortical lesions, and WM lesions), and mean and median values were computed for R_1_, MTRasym_corr_, MTR_RexCorr_, and AREX_corr_ in these regions of interest for each participant.

### Statistical Analysis

A two-sample t-test was performed to test differences in demographic variables, cognitive test scores, and CEST contrast indices (for each tissue region of interest) between healthy volunteers and MS patients. Within each group, a paired *t*-test was performed to determine whether CEST indices differed between GM and WM. CEST contrast in white matter lesions and cortical lesions was also compared to the corresponding normal-appearing tissue in MS patients (paired *t*-test) and to WM and GM in healthy volunteers (two-sample *t*-test). In patients with MS, associations between MT-corrected AREX contrast, R_1_, and clinical measures of disease status and cognitive function were investigated using a Spearman partial correlation with age, sex, and years of education included as covariates since these variables can influence performance on several cognitive tests. Additionally, some studies have shown age and sex differences in glutamate levels in the brain (56–58). Associations between AREX contrast and cognitive test scores were not examined in the healthy group because variance within physiologic levels of glutamate is expected to be highly regulated and unrelated to cognition in otherwise healthy individuals. Given the exploratory nature of this study to examine glutamate-weighted AREX contrast in MS, no correction for multiple comparisons was employed and *t*-test results are reported with cutoff levels of 0.05, 0.01, and 0.001 for comparisons between tissue regions.

## Results

### Isolating CEST Effects

**Figure 1:**
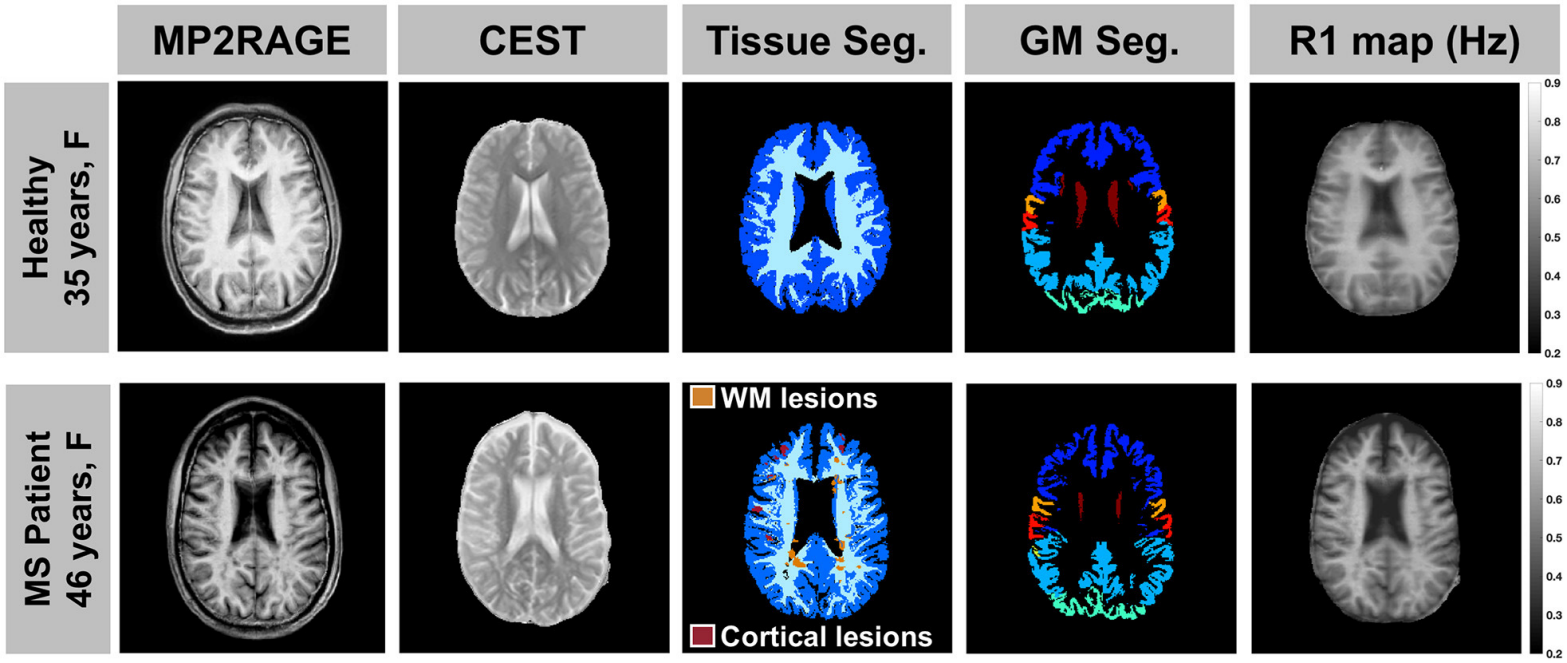
A representative anatomical MP2RAGE image, CEST image (individual dynamic at Δω = −5.0 ppm normalized to the unsaturated S_0_ data), tissue segmentation, and R_1_ map are shown for a healthy volunteer (top: 35-year-old female) and a patient with MS (bottom: 46-year-old female, EDSS = 2, duration = 1 year). All image volumes were registered to the 10 mm-thick CEST slice.

Glutamate-weighted CEST and anatomical images were acquired in all participants without adverse events. In one MS patient we did not complete the inversion recovery sequence, resulting in a missing R_1_ map for the AREX correction. CEST data from a different MS patient and one healthy volunteer were excluded from analyses due to motion artifact-related noise. The resulting data set for CEST analyses with the AREX quantification method consisted of 19 healthy volunteers and 18 MS patients. Representative anatomical images, CEST images (normalized individual dynamic at Δω = −5.0 ppm), tissue segmentation, and R_1_ maps are shown for a healthy volunteer and a patient with MS in Figure 1. Anatomical images, segmentation masks, and R_1_ maps were all registered to the 10 mm-thick CEST slice.

**Figure 2:**
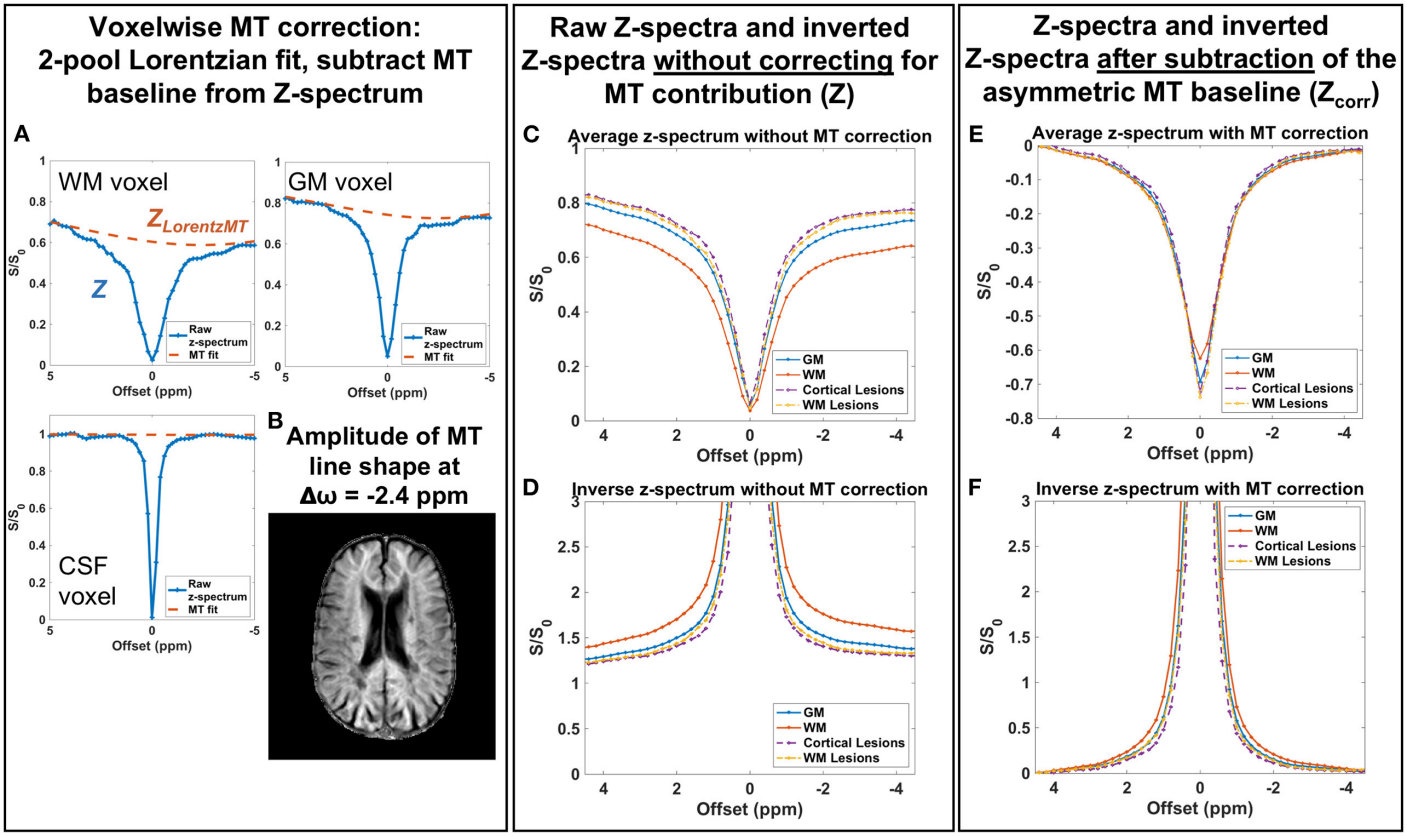
**(A)** A 2-pool Lorentzian model was fit to the measured z-spectrum (Z) in each voxel and the resulting MT Lorentzian component (Z_LorentzMT_) is shown for representative voxels in different tissues. **(B)** The amplitude of the MT Lorentzian line shape at −2.4 ppm shows little to no MT contribution in CSF and the largest MT contribution in white matter. **(C,D)** Raw z-spectra and inverted z-spectra without the MT correction show differences due to broad MT saturation effects. **(E,F)** After subtracting the MT baseline, differences between gray and white matter z-spectra and inverted spectra due to MT effects are minimized. Example spectra shown are from a patient with MS (46-year-old male, EDSS = 4, duration = 16 years).

To isolate CEST effects from the confounding effects of MT, direct saturation, and T_1_ changes, we first obtained the MT baseline for each voxel using a 2-pool Lorentzian fit (MT and DS pools), then subtracted the MT spectrum from the measured CEST z-spectrum. Examples of the MT baseline in different tissues as well as the resulting impact on the average z-spectra and inverse z-spectra for GM, WM, and lesions are shown in Figure 2. WM voxels show the greatest influence from asymmetric MT effects on the CEST z-spectra, while GM has a smaller MT influence and CSF has little to no MT contribution, as expected (Figures 2A,B). After subtracting the MT baseline, the tissue differences and broad asymmetry in the z-spectra and inverse z-spectra are reduced (Figures 2C–F).

**Figure 3:**
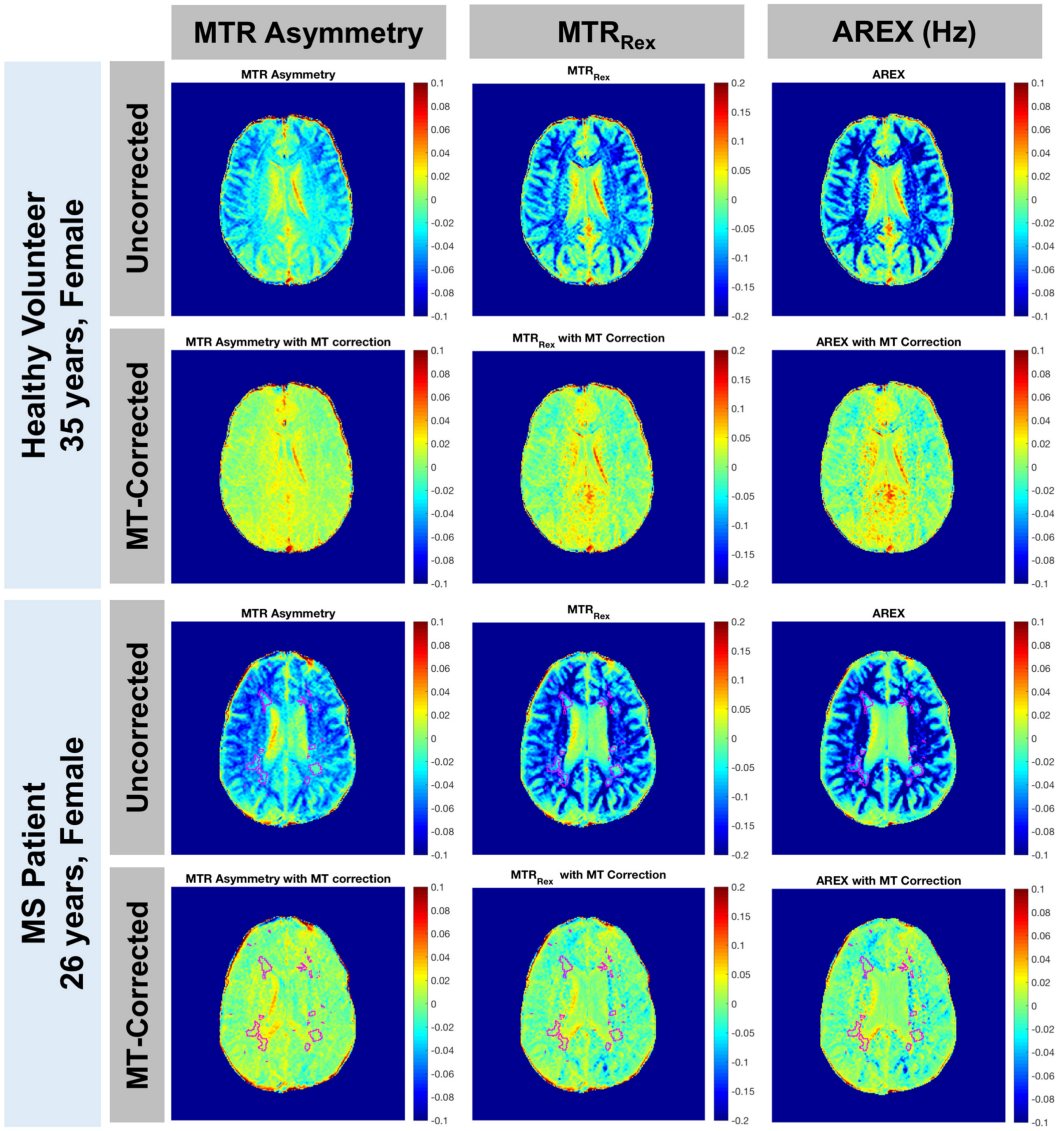
MTR asymmetry, MTR_Rex_, and AREX maps for Δω = 3.0 ppm are shown with and without the MT baseline removal for a representative healthy volunteer (rows 1 and 2: 35-year-old female) and a patient with MS (rows 3 and 4: 26-year-old female, EDSS = 0, duration = 6 years). Before removing the MT contribution, there is a greater difference between GM and WM for all 3 CEST indices (rows 1 and 3). After removal of the asymmetric effect, CEST contrast shifts toward positive values and the difference between GM and WM is reduced but still present (rows 2 and 4). Lesions are outlined in the patient images.

**Figure 4:**
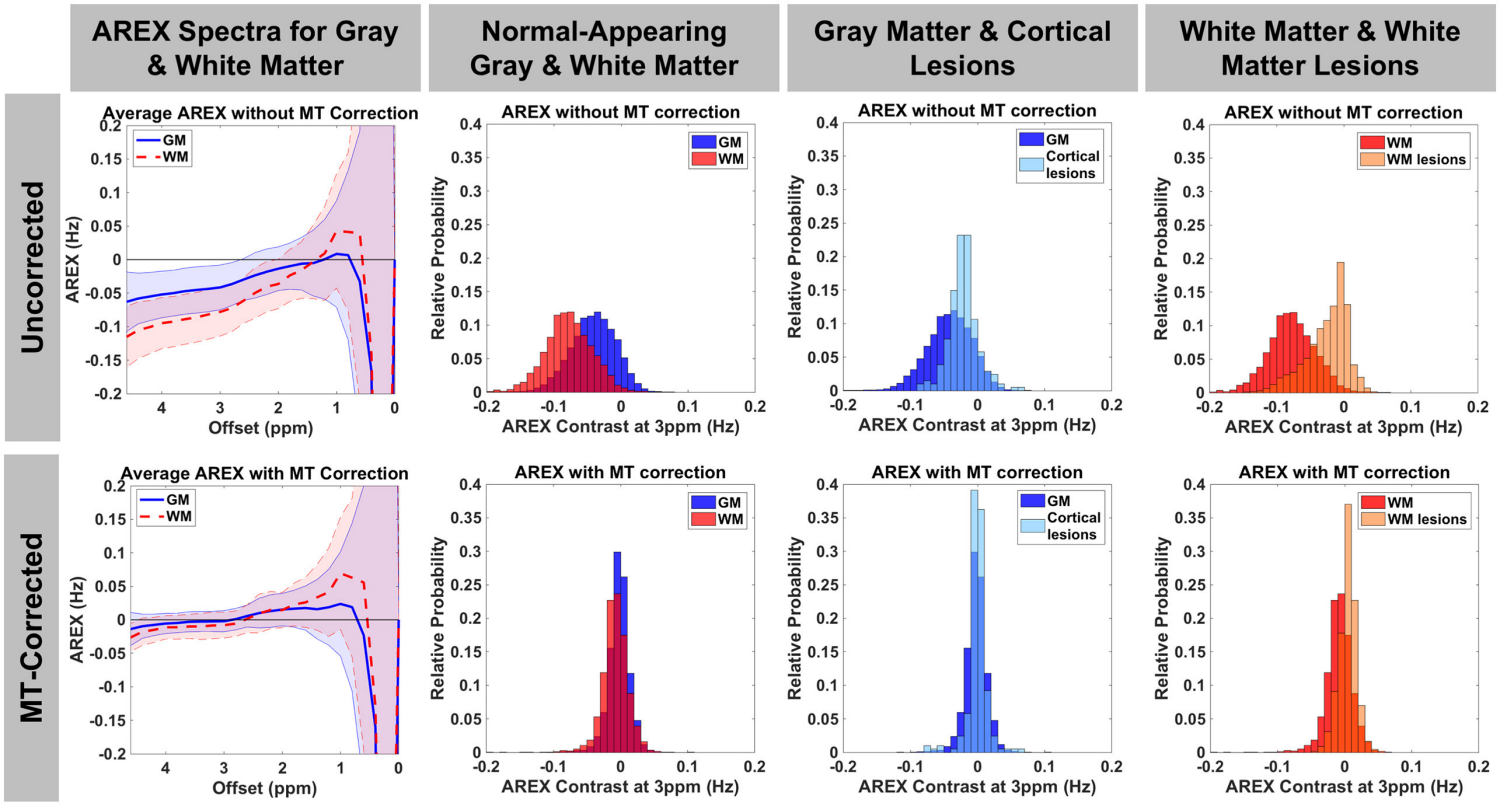
AREX spectra and histograms for AREX contrast at Δω=3.0 ppm are shown without (top row) and with (bottom row) MT baseline removal for a representative patient with MS (46-year-old male, EDSS = 4, duration = 16 years). Before removing the MT contribution, there is a greater difference between GM and WM and the majority of the AREX values are negative. After removal of the asymmetric MT effect, AREX contrast shifts more toward positive values and the difference between GM and WM is reduced, but slightly higher AREX in GM remains as expected. After correction for MT effects, AREX in cortical lesions overlaps with that in GM, but WM lesions still show a slight increase in AREX relative to WM.

MTRasym, MTR_Rex_, and AREX maps were calculated using Equations 6, 8, 9 with Δω = 3.0 ppm to target glutamate. Representative maps with and without the MT baseline subtraction are shown in Figure 3. As expected, the CEST indices show larger contrast between GM and WM due to the greater myelin content in WM when the MT influence on the measured CEST spectra is not removed. Additionally, the asymmetric MT baseline with a peak around Δω = −2.4 ppm results in more negative values in the observed CEST asymmetry. After removal of the MT baseline, the difference between GM and WM is visibly reduced but not eliminated, and the values are shifted toward positive values. Quantification of AREX contrast without and with MT baseline removal shows the same effects with data from a representative patient shown in Figure 4. The mean AREX contrast in GM and WM shifts toward positive values and the difference between the tissues is reduced after removal of the MT influence. In this patient, cortical lesions do not show a difference in AREX contrast relative to NAGM after isolating the CEST effects from MT, direction saturation, and T_1_ contributions, but there is a slight increase in AREX contrast in WM lesions relative to NAWM.

### Group Differences in CEST Indices

**Figure 5:**
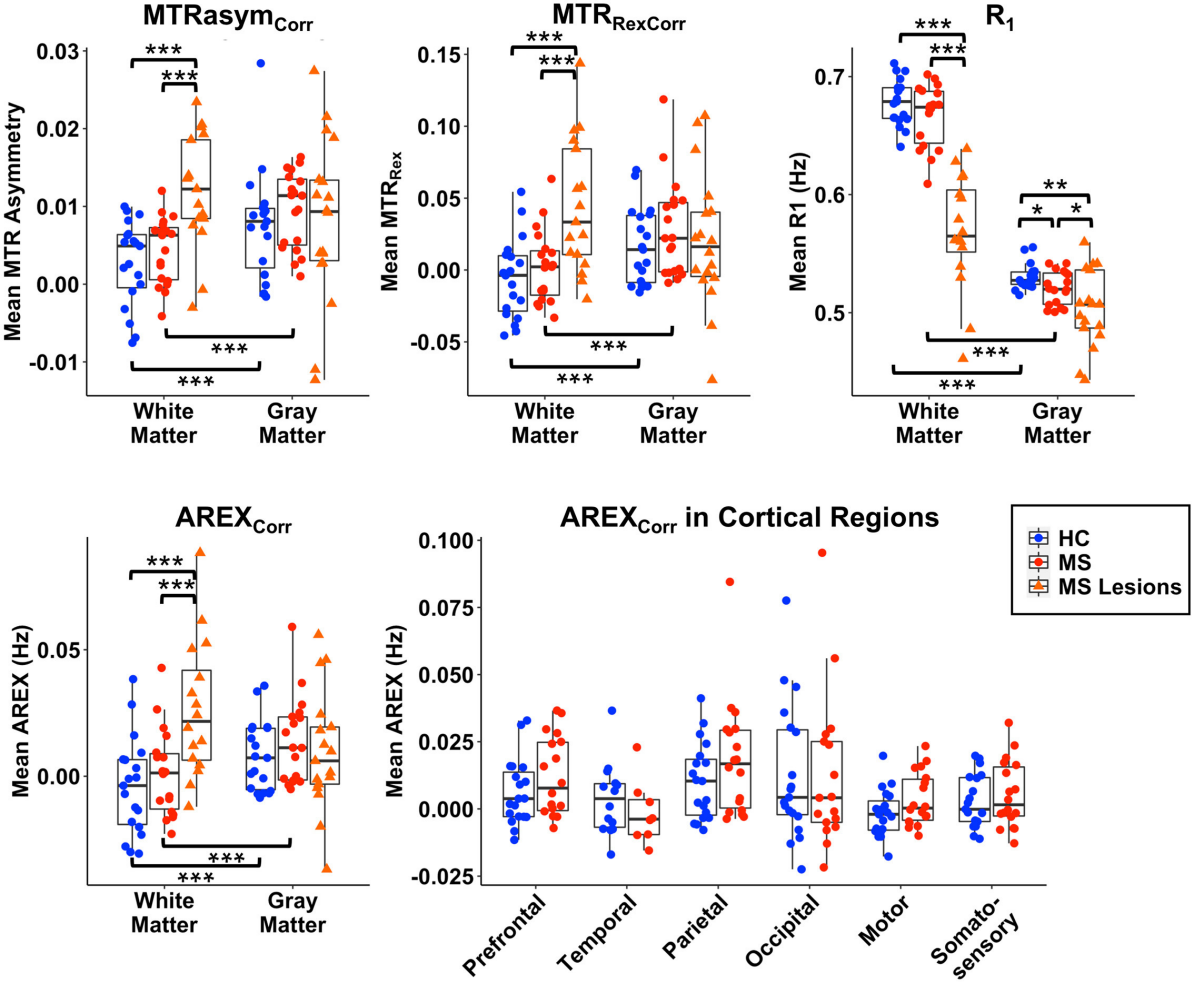
Mean values of MTRasym_Corr_, MTR_RexCorr_, AREX_Corr_, and R_1_ are shown for WM, GM, WM lesions, and cortical lesions for group comparisons. AREX_Corr_ values are also shown for each cortical GM region. WM lesion values differ significantly from normal-appearing WM in MS patients and from healthy control (HC) WM for all CEST-derived indices and for R_1_. Significant differences between groups in GM and cortical lesions are only present in R_1_. No group differences are significant for AREX_Corr_ within cortical regions. All indices differ significantly between GM and WM within each group. *p < 0.05, **p < 0.01, ***p < 0.001.

Mean and median values for R_1_ and MT-corrected CEST indices in WM, GM, white matter lesions, and cortical lesions are shown in Figure 5 and Supplementary Figure 1, respectively. Within the healthy volunteer and MS patient groups, MTRasym_Corr_, MTR_RexCorr_, and AREX_Corr_ values at Δω = 3.0 ppm were significantly greater in GM than in WM (*p* < 0.001). CEST indices in NAGM and NAWM in MS patients did not differ significantly from healthy GM and WM in the control group, but all CEST indices were significantly elevated in WM lesions relative to NAWM and healthy WM (*p* < 0.001). R_1_ differed significantly between WM and GM within each group, and R_1_ was significantly decreased in WM lesions relative to NAWM and healthy WM (*p* < 0.001). NAGM and cortical lesions in MS patients also had significant reductions in R_1_. When the cortex was subdivided into regions, there were no significant differences in AREX_Corr_ between MS patients and healthy volunteers. The distributions of AREX_Corr_ values in each group and tissue region of interest (ROI) are presented with the average histogram and standard deviation for each group/tissue in Supplementary Figure 2. The distributions were similar between healthy volunteers and MS patients for NAGM and NAWM, while lesion histograms show greater inter-subject variability. AREX_Corr_ in WM lesions clearly shifted toward higher values.

### Correlations Between MRI Indices, Disability, and Cognitive Function

**Figure 6:**
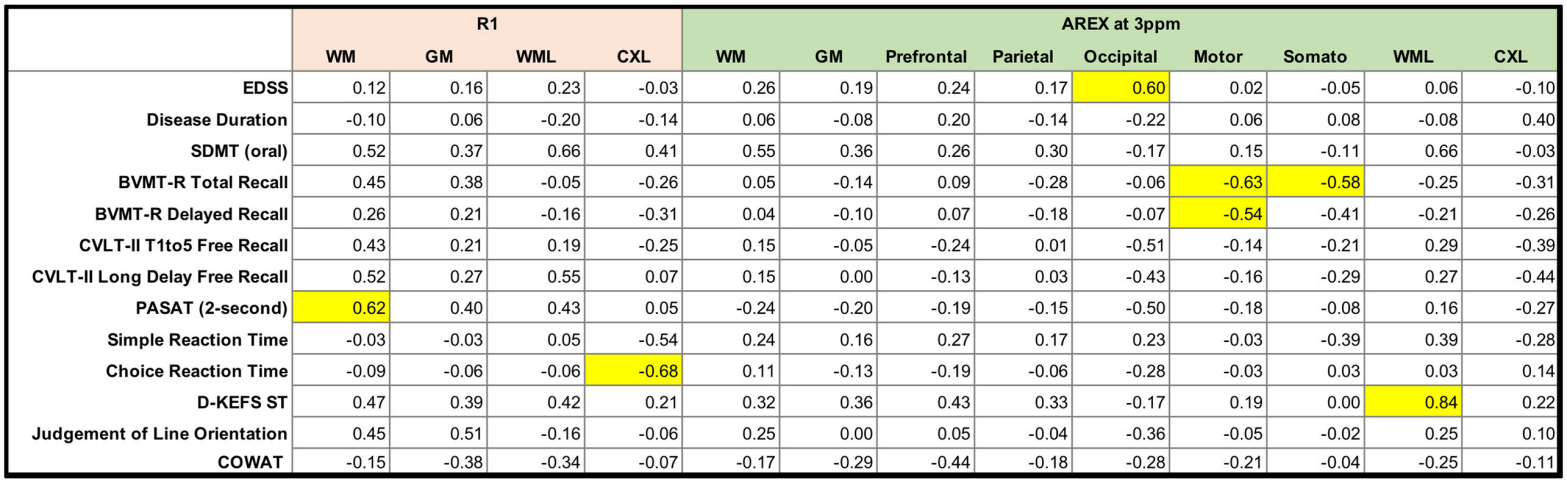
Matrix of Spearman rho values for partial correlations between clinical and cognitive variables and mean R_1_ and AREX values in patients with MS. Age, education, and sex were included as covariates. Correlations are highlighted in yellow if p < 0.05. SDMT = Symbol Digit Modalities Test; BVMT-R = Brief Visuospatial Memory Test-Revised; CVLT-II = California Verbal Learning Test, 2nd edition; PASAT = Paced Auditory Serial Addition Test; D-KEFS ST = Delis-Kaplan Executive Function System Sorting Test; COWAT = Controlled Oral Word Association Test.

Group differences in measures of cognitive function are reported in Table 3. Significant differences were observed for the California Verbal Learning Test 2^nd^ edition total free recall (*p* < 0.001) and long delay free recall (*p* < 0.01), Symbol Digit Modalities Test (*p* < 0.001), Brief Visuospatial Memory Test-Revised delayed recall (*p* < 0.05), Controlled Oral Word Association Test (*p* < 0.001), and Simple Reaction Time (*p* < 0.05). Associations between R_1_ and AREX_Corr_ MRI indices and EDSS score, disease duration, and cognitive function in patients with MS were examined using a Spearman partial correlation analysis with age, sex, and education as covariates. The resulting Spearman rho values are shown in Figure 6, with significant correlations highlighted in yellow (*p* < 0.05). Mean R_1_ values in MS NAWM correlated positively with 2-s PASAT scores (rho = 0.62), and R_1_ values in cortical lesions correlated negatively with Choice Reaction Time (rho = −0.68). EDSS scores correlated positively with mean AREX_Corr_ values in the occipital cortex (rho = 0.60). Significant negative correlations were detected between visuospatial memory (BVMT-R) scores and mean AREX_Corr_ values in the motor and somatosensory cortices (rho values of −0.63, −0.54, and −0.58). Finally, mean AREX_Corr_ in WM lesions correlated positively with scores on the D-KEFS Sorting Test (rho = 0.84). AREX_Corr_ and R_1_ values within each tissue ROI were not correlated with each other (Supplementary Figure 3).

**Table 3:** Measures of cognitive function.

**Cognitive assessment, mean ±SD**	**MS patients (*n* = 15)**	**Healthy volunteers (*n* = 20)**
CVLT-II (trials 1–5 total free recall)	44.7 ± 9.1^***^	56.9 ± 9.9
CVLT-II (long delay free recall)	9.2 ± 3.2^**^	12.8 ± 2.9
SDMT (oral)	52.7 ± 12.2^***^	65.5 ± 8.1
BVMT-R (total recall)	24.3 ± 6.1^†^	27.4 ± 4.1
BVMT-R (delayed recall)	8.8 ± 2.6^†^^*^	10.5 ± 1.3
PASAT (2-second version)	33.2 ± 9.2	38.0 ± 12.3
D-KEFS ST (total correct, set 1 + set 2)	9.7 ± 2.5	10.9 ± 2.6
Judgement of line orientation	25.2 ± 4.3	26.2 ± 3.1
COWAT (total words, C + F + L)	34.0 ± 7.1^***^	45.6 ± 9.0
Simple reaction time	295.9 ± 46.1 ms^†^^*^	266.4 ± 32.4 ms
Choice reaction time	499.0 ± 91.4 ms^†^	452.8 ± 85.1 ms

*15 of the 20 patients with MS and 19 of the 20 healthy volunteers completed the Minimal Assessment of Cognitive Function in MS (MACFIMS) battery. The remaining 5 patients with MS completed a subset of the tests*.

*MS, multiple sclerosis; CVLT-II, California Verbal Learning Test, 2^nd^ edition; SDMT, Symbol Digit Modalities Test; BVMT-R, Brief Visuospatial Memory Test-Revised; PASAT, Paced Auditory Serial Addition Test; D-KEFS ST, Delis-Kaplan Executive Function System Sorting Test; COWAT, Controlled Oral Word Association Test*.

* *p < 0.05*,

** *p < 0.01*,

*** *p < 0.001: Patients with MS differ significantly from healthy volunteers*.

† *MS patients n = 20*.

## Discussion

In this study, glutamate-weighted CEST MRI was applied at 7.0 T field strength with corrections for the confounding influences of MT, spillover, and T_1_ effects. We first demonstrated the effect of removing the asymmetric MT baseline from CEST z-spectra, then sought to assess whether glutamate-sensitive, MT-corrected AREX contrast is sensitive to pathology in the brain in MS by (1) comparing AREX in MS lesions, NAGM, and NAWM to healthy GM and WM, and (2) exploring correlations between AREX and measures of disease status and cognitive impairment.

### Effects of MT and AREX Corrections on Glutamate-Weighted CEST

We found that fitting the CEST z-spectrum with a Lorentzian line shape removes the broad asymmetry that is attributed to MT from the semisolid macromolecular components within tissue (e.g., myelin) (Figure 2). Prior studies of GluCEST in the brain have relied on an asymmetry analysis for quantification of CEST contrast (Equation 2) (13, 15, 16, 59, 60), which does not completely isolate the CEST effects of metabolites from competing effects, especially when high irradiation powers are applied. Because MS is a demyelinating disease, it is important to address the influence of myelin changes (46–48) and its impact on the CEST spectrum to more selectively study metabolic changes contributing to disease pathology. Lorentzian fitting approaches for removing the MT component were recently demonstrated for GluCEST in the healthy human brain at 7.0 T (45), in the healthy rat brain at 9.4T (14), and in the rat brain with a tumor at 9.4T (45). As in the current work, these prior studies found that removal of the MT baseline resulted in a decrease in the difference in CEST signal between GM and WM. Our results show that although the quantitative CEST indices (MTRasym, MTR_Rex_, and AREX) have less GM:WM contrast after MT correction, the average CEST signal at 3.0 ppm in GM is still significantly greater than that in WM (Figures 4, 5). This result is in agreement with MT-corrected GluCEST applied in healthy volunteers at 7.0 T by Debnath et al. (45) and is presumed to be due to the higher concentration of glutamate in GM. Cui et al. also found that MTRasym at 3.0 ppm remained significantly greater in GM than in WM in the rat brain after the MT correction, but this tissue difference did not persist for AREX quantification and there was no difference in water T_1_ between GM and WM (14). In our study, we observed differences in R_1_ (1/T_1_) between GM, WM, and lesions (Figure 5) which correspond with prior literature and support the need for the AREX correction to produce CEST contrast more reflective of the metabolite of interest (30). Cui et al. also performed phantom experiments to confirm that the CEST signal from glutamate at 3.0 ppm dominates that from other brain metabolites, and they used rat brain tissue homogenate dialysis experiments to show that amine protons on protein lysine residues also contribute significantly to CEST signal at 3.0 ppm (14). Potential causes for the variability between our MT-corrected CEST results and these prior studies include differences in cohort (human or rat), field strength, CEST saturation parameters, and CEST quantification method applied after the MT baseline removal [conventional GluCEST equation (45) or AREX (14)].

In this work, we used the MT-corrected signal at the opposite frequency (−3.0 ppm) as the reference for computing MTRasym and AREX contrast at the glutamate resonance frequency of 3.0 ppm. Without MT correction, these signals are contaminated by MT effects which are broad and peak near −2.4 ppm. After removal of the MT baseline, it is still possible that our reference signal could be confounded by relayed nuclear Overhauser enhancement (rNOE) effects which are centered around −3.5 ppm but range from −2 to −5 ppm (28, 44, 45). Some prior AREX studies have employed a 5-pool multi-Lorentzian fitting approach to quantify the amplitude of a given CEST peak (30, 61). This approach is especially useful for amide proton transfer (APT)-weighted CEST since that frequency of interest is 3.5 ppm and use of the signal at −3.5 ppm as a reference would be confounded by rNOE effects. This approach requires estimating peak frequencies, amplitudes, and widths for each pool and has been shown to work well for CEST data with high spectral resolution. In the current study, the signal at −3.0 ppm was chosen as the reference for quantifying glutamate-weighted CEST contrast since high saturation powers (e.g., 2.9 μT and higher) have been shown to attenuate the rNOE contributions to the z-spectra in both GM and WM (45, 62), and our nominal saturation power was 4.25 μT. While greater saturation pulse power is necessary for targeting protons with faster exchange rates such as the amine protons on glutamate, future studies could examine the tradeoffs between the increase in spectral resolution and the decrease in sensitivity to glutamate that would occur when decreasing the saturation pulse B_1_ amplitude.

### CEST Signal Differs in WM Lesions Independently of Changes in MT and R_1_

Changes in glutamate homeostasis have been linked to the pathophysiology of disease progression in MS by prior studies in animal models (21, 63–65) and MRS studies in MS patients (6–8, 66–68). Aspects of glutamate involvement in MS pathology include increased extracellular glutamate leading to excitotoxicity and neuronal and glial cell death, increased production of glutamate in macrophages and microglial cells in WM lesions (via elevation of glutaminase expression), and deficient glutamate reuptake by oligodendrocytes in MS WM (7, 69, 70).

After MT correction, we did not find any significant differences in CEST indices (MTRasym_Corr_, MTR_RexCorr_, AREX_Corr_) between NAGM or NAWM in patients with MS and the corresponding healthy GM or WM in healthy volunteers (Figure 5 and Supplementary Figure 1). The significant decrease in R_1_ observed in the NAGM of MS patients relative to healthy GM, and in GM and WM lesions relative to normal-appearing and healthy tissues, support the importance of AREX calculation to remove confounding R_1_ effects from the observed CEST signal. After applying the AREX correction, we confirmed that AREX_Corr_ values did not correlate with R_1_ values in each tissue region (Supplementary Figure 3), indicating that AREX_Corr_ is independent of R_1_ differences. Our findings for R_1_ agree with prior studies showing lower R_1_ in WM lesions and in NAGM (46, 71, 72). Additionally, the decrease in GM:WM tissue contrast in CEST indices after removal of MT highlights the influence of MT effects on the quantified CEST signal (Figure 4) and corresponds with recent literature (45).

In a prior study, we found increased GluCEST contrast in the prefrontal cortex of RRMS patients compared to healthy volunteers; however, in that work, we implemented the conventional GluCEST quantification method and did not apply MT removal or R_1_ corrections (16). In the current study, we observed non-significant trends toward increased GM AREX_Corr_ in the prefrontal and parietal cortices of MS patients (Figure 5). Discrepancies in findings could be due to the influences of underlying myelin and R_1_ (71) changes that co-occur in MS pathology, and/or the use of a different cohort of MS patients. Reported glutamate abnormalities in MS GM have also varied in the MRS literature, and include no significant difference compared to controls (7), lower glutamate values in the cingulate and parietal cortices (8), and lower values in parietal and sensorimotor regions (68).

Although we did not detect differences in normal-appearing tissues in patients, we found that after removing the MT, spillover, and R_1_ contributions, AREX_Corr_ in WM lesions was significantly increased relative to NAWM and healthy WM (Figure 5 and Supplementary Figures 1, 2). To our knowledge, glutamate-weighted CEST has not been evaluated in MS patients in studies other than our prior work, but APT-weighted CEST has been explored in MS. In a preliminary study of four MS patients, Dula et al. noted that APT-weighted CEST asymmetry within heterogeneous lesions could either increase or decrease relative to healthy WM (18). Another APT-weighted CEST study with a larger sample size of 27 relapsing-remitting and secondary progressive MS patients found that mean APT-weighted CEST signal intensity increased in MS lesions relative to healthy WM (17). Finally, an APT-weighted CEST study in patients with amnestic mild cognitive impairment (not MS) also found higher CEST signals in patients relative to healthy controls in several regions of the brain, including the GM and WM in the occipital and temporal lobes (73).

Although these prior CEST studies in MS and amnestic cognitive impairment quantify the CEST signal at 3.5 ppm (attributed to amide protons on endogenous proteins and peptides), the glutamate-weighted CEST signal at 3.0 ppm could also be influenced by amine protons on protein lysine residues as shown by *in vitro* experiments (14). We expect that glutamate is the primary contributor to the MT-corrected AREX_Corr_ signal, since greater signal is observed in GM which has a higher glutamate concentration than WM. Our finding of increased glutamate-weighted AREX_Corr_ in WM lesions also corresponds with MR spectroscopy studies that found evidence of glutamate toxicity in MS patients via elevated glutamate in acute WM lesions (6) and in NAWM (7). Elevated glutamate in active WM lesions is likely caused by inflammatory infiltrates with excess glutamate released by activated leukocytes, macrophages, and microglial cells (6), along with impaired glutamate uptake by oligodendrocytes (70). Srinivasan et al. did not find elevated glutamate in chronic WM lesions (6), which may explain the greater inter-subject variability in glutamate-weighted AREX_Corr_ signal of WM lesions relative to NAWM and healthy WM in our study (Figure 5 and Supplementary Figures 1, 2), as we did not distinguish active from chronic lesions.

### AREX_Corr_ and R_1_ Values Are Associated With Disease Status and Cognitive Function

The patient cohort in this study presented with mild cognitive impairment compared to the matched healthy cohort, with significantly lower scores on some of the tests administered and scores within normal limits on others (74–78). Lower performance for the patient cohort was observed in the domains of verbal and visuospatial learning and memory, information processing speed, verbal fluency, and cognitive flexibility; thus, we explored whether glutamate-sensitive CEST contrast and R_1_ were associated with cognitive function.

Despite multiple MRS studies showing increased glutamate in WM lesions and NAWM and decreased glutamate in NAGM, findings on the relationships between brain glutamate concentration and clinical outcomes in MS have varied. Azevedo et al. found that elevated glutamate alone in NAWM did not predict clinical measures of disability in a longitudinal study; however, an elevated glutamate/*N*-acetylaspartate ratio in NAWM predicted a decline in brain volume and worsening clinical disability measures (MS Functional Composite, EDSS, and PASAT). Here, we did not find any significant correlations between AREX_Corr_ in NAWM and measures of cognitive function or disease status (disease duration or EDSS), but we observed a significant positive correlation between AREX_Corr_ in WM lesions and performance on the D-KEFS Sorting Test (Figure 6). The D-KEFS Sorting Test assesses executive function (e.g., categorization, abstraction, flexibility of thinking), a cognitive domain commonly affected in MS (79). Based on the significant increase in AREX_Corr_ in WM lesions relative to healthy WM, we might expect that higher AREX_Corr_ values would instead correspond with lower test scores. Although D-KEFS Sorting Test scores were lower in the patient group, the deficit was not significant and the patient group mean fell within normal limits (77). The observed association may also be confounded by not distinguishing active from chronic lesions.

Results from prior studies of GM glutamate levels relating to cognitive outcomes in MS are also mixed. An MRS study using large single-voxel measurements (ranging from 6.7 to 15.6 mL in volume) found that poor visuospatial memory performance was significantly associated with lower glutamate concentration in the hippocampus, thalamus, and cingulate cortex of MS patients but not in healthy controls (8), even after correcting for magnetization ratio. In contrast, another single-voxel MRS study initially found that parietal glutamate was a significant predictor of PASAT and SDMT performance; however, this relationship was no longer significant after adjusting for MT ratio, *N*-acetylaspartate level, and normalized brain volume (markers of structural damage) (68). In our prior GluCEST study, we found that increased GluCEST contrast in the prefrontal cortex was significantly correlated with SDMT scores and Choice Reaction Time, both measures of information processing speed (16), although that analysis did not include corrections for MT and R_1_. In the current study, we found that glutamate-weighted AREX_Corr_ in the occipital cortex was positively correlated with EDSS scores. Other significant relationships between GM AREX_Corr_ and cognition included negative correlations between AREX_Corr_ in the motor and somatosensory cortices and BVMT-R scores, a measure of visuospatial learning and memory. The direction of these correlations also corresponds with our prior study in which higher glutamate in the cortex was associated with worse cognitive performance and increased disability (16). However, we would not typically expect these particular cortical regions to be associated with a test of visuospatial memory, and it is possible that this observed deficit is related to overall brain pathology rather than directly related to abnormal local glutamate levels. Additionally, while BVMT-R scores were lower in this patient cohort relative to the matched healthy cohort, the difference was only statistically significant in the delayed component of the test and the MS group mean was within normal limits for the BVMT-R (78).

We also examined correlations between R_1_ and cognition in our patient cohort because R_1_ measurements are notably sensitive to, but unspecific for, several pathological changes. Lower R_1_ in NAWM and in cortical lesions are documented features of MS (71, 72, 80–83). Decreased R_1_ can reflect increased water content, gliosis, and myelin and axonal loss, all known to occur diffusively in the NAWM. Damage to the WM in various tracts is known to be associated with cognitive impairment in several domains, mainly information processing, attention, and executive function (84–86). Cortical lesion burden is linked with disability and overall cognitive impairment, independently of subcortical WM damage (87, 88). Here, we found that higher R_1_ in the NAWM was significantly correlated with PASAT score, while higher R_1_ in cortical lesions was correlated with faster Choice Reaction Time. A high PASAT score and a faster Choice Reaction Time are both indices of good performance. Thus, detection of associations between worse cognitive scores and tissue alterations measured with R_1_ in different tissue compartments is an expected phenomenon and consistent with the expected associations between disease burden in the brain and cognition.

### Limitations and Conclusions

Some limitations of this study include the modest sample size of MS patients with complete cognitive battery data, which limited our ability to detect independent effects of different CEST MRI-derived variables given the number of covariates (e.g., age, sex, education), and the mild cognitive impairment status of these patients (several test scores within normal limits). Additionally, we did not correct for atrophy in our analyses because atrophy is a global process in the brain in MS, and the CEST data represented a 10 mm-thick slice. A coarse slice thickness was chosen to increase the signal-to-noise ratio (SNR) of CEST which is typically a low-SNR acquisition, but this low through-plane resolution could cause some partial volume contamination, especially for small lesions. Future studies could consider 3D acquisitions or other faster imaging methods to improve both in-plane and through-plane resolution. We did not separate active lesions from chronic lesions since no gadolinium contrast was used in this study, but future studies of glutamate in MS lesions may benefit from a separation of lesion type/status since different pathological processes could result in increased or decreased glutamate within an individual patient or patient cohort. In terms of glutamate-weighted CEST signal quantification, we chose to use −3.0 ppm as the reference frequency for the signal at 3.0 ppm, which could potentially be affected by rNOE effects as described above despite the sequence being optimized for sensitivity to glutamate CEST effects. We used a relatively simple two-pool Lorentzian model to fit the broad MT baseline in our CEST data and it performed well, but future studies could also employ higher spectral resolution for z-spectra and acquire offset frequencies outside the typical CEST window of −5.0–5.0 ppm to take advantage of other strategies for fitting CEST z-spectrum components (30, 61) and modeling the macromolecular MT effects that confound CEST quantification (22). Future work may also benefit from using an 8-channel parallel transmit system at 7.0 T to improve the homogeneity of the B_1_+ field for CEST saturation pulses. Finally, we did not correct for multiple comparisons in our analyses to avoid overcorrecting in this exploratory study. With a larger sample size and correction for multiple correlations, the strength of the associations observed in the current cohort may change. In Figure 5, there were 38 *t*-tests performed for all variables combined; with a Bonferroni correction, the significance level would be *p* < 0.0013 and the differences observed between WM lesions and healthy WM and between WM and GM within each group remain significant (*p* < 0.001). The correlations observed in Figure 6 would not remain significant after a correction for multiple comparisons given the current sample size and number of variables.

Despite the limitations of this study, our results highlight the utility of glutamate-weighted CEST at ultrahigh field as a metabolic imaging technique to probe the pathology underlying clinical symptoms, including cognitive impairment, in neurological diseases such as MS, and we demonstrated the importance of isolating the glutamate-weighted CEST signal from confounding factors such as changes in R_1_ and macromolecular MT effects that occur simultaneously in demyelinating, inflammatory diseases.

## Data Availability Statement

The raw data supporting the conclusions of this article will be made available by the authors, without undue reservation.

## Ethics Statement

The studies involving human participants were reviewed and approved by Vanderbilt University Institutional Review Board. The patients/participants provided their written informed consent to participate in this study.

## Author Contributions

KO'G, FB, HW, and SSm designed the study and interpreted the results. KO'G, SSa, QO, BB, and HF performed the experiments. KO'G, SSa, SC, AC, and BR contributed to image processing and data analysis. RL, RD, BR, and SSm provided technical advice. FB, AS, SM, JN, and KY performed and confirmed segmentation of lesions. KO'G, AC, and SSm wrote the paper. All authors contributed constructively to the manuscript.

## Funding

Research reported in this publication was supported in part by funding from the National Institutes of Health (NINDS award numbers F32NS101788, R01NS109114, and R21NS116434-01A1, NCATS Vanderbilt CTSA award number UL1TR000445, NCATS award number KL2TR002245, and NIBIB award number K01EB030039), the U.S. Department of Defense (award number W81XWH-13-1-0073), the National Multiple Sclerosis Society (award numbers RG-1501-02840 and RG-1901-33190), the Conrad Hilton Foundation, the Veterans Health Administration (award number I01CX002160-01A1) and the VUMC Faculty Research Scholars program. The content is solely the responsibility of the authors and does not necessarily represent the official views of the National Institutes of Health. The authors declare that FB received funding from Biogen Idec. The funder was not involved in the study design, collection, analysis, interpretation of data, the writing of this article or the decision to submit it for publication.

## Conflict of Interest

The authors declare that the research was conducted in the absence of any commercial or financial relationships that could be construed as a potential conflict of interest.

## Publisher's Note

All claims expressed in this article are solely those of the authors and do not necessarily represent those of their affiliated organizations, or those of the publisher, the editors and the reviewers. Any product that may be evaluated in this article, or claim that may be made by its manufacturer, is not guaranteed or endorsed by the publisher.

## Abbreviations

CEST: chemical exchange saturation transfer
AREX: apparent exchange-dependent relaxation
MTRasym: magnetization transfer ratio asymmetry
MTR_Rex_: magnetization transfer ratio employing inverse Z-spectrum
MT: magnetization transfer
MP2RAGE: magnetization prepared 2 rapid acquisition gradient echoes
MP-FLAIR: magnetization prepared fluid-attenuated inversion recovery
TFE: Turbo Field Echo
TSE: Turbo Spin Echo
SENSE: sensitivity encoding
GM: gray matter
WM: white matter
MS: multiple sclerosis
EDSS: Expanded Disability Status Scale
WRAT4: Wide Range Achievement Test 4^th^ edition
CVLT-II: California Verbal Learning Test 2^nd^ edition
SDMT: Symbol Digit Modalities Test
BVMT-R: Brief Visuospatial Memory Test-Revised
PASAT: Paced Auditory Serial Addition Test
D-KEFS ST: Delis-Kaplan Executive Function System Sorting Test
COWAT: Controlled Oral Word Association Test.
