# Supplementary material for: Relaxation-Compensated Chemical Exchange Saturation Transfer MRI in the Brain at 7T: Application in Relapsing-Remitting Multiple Sclerosis
Source: Front Neurol. 2022 Feb 25;13:764690. doi: 10.3389/fneur.2022.764690 (PMC8923037; doi:10.3389/fneur.2022.764690)
Supplement: Supplementary file 1 [file Data_Sheet_1.PDF]

Supplementary Material

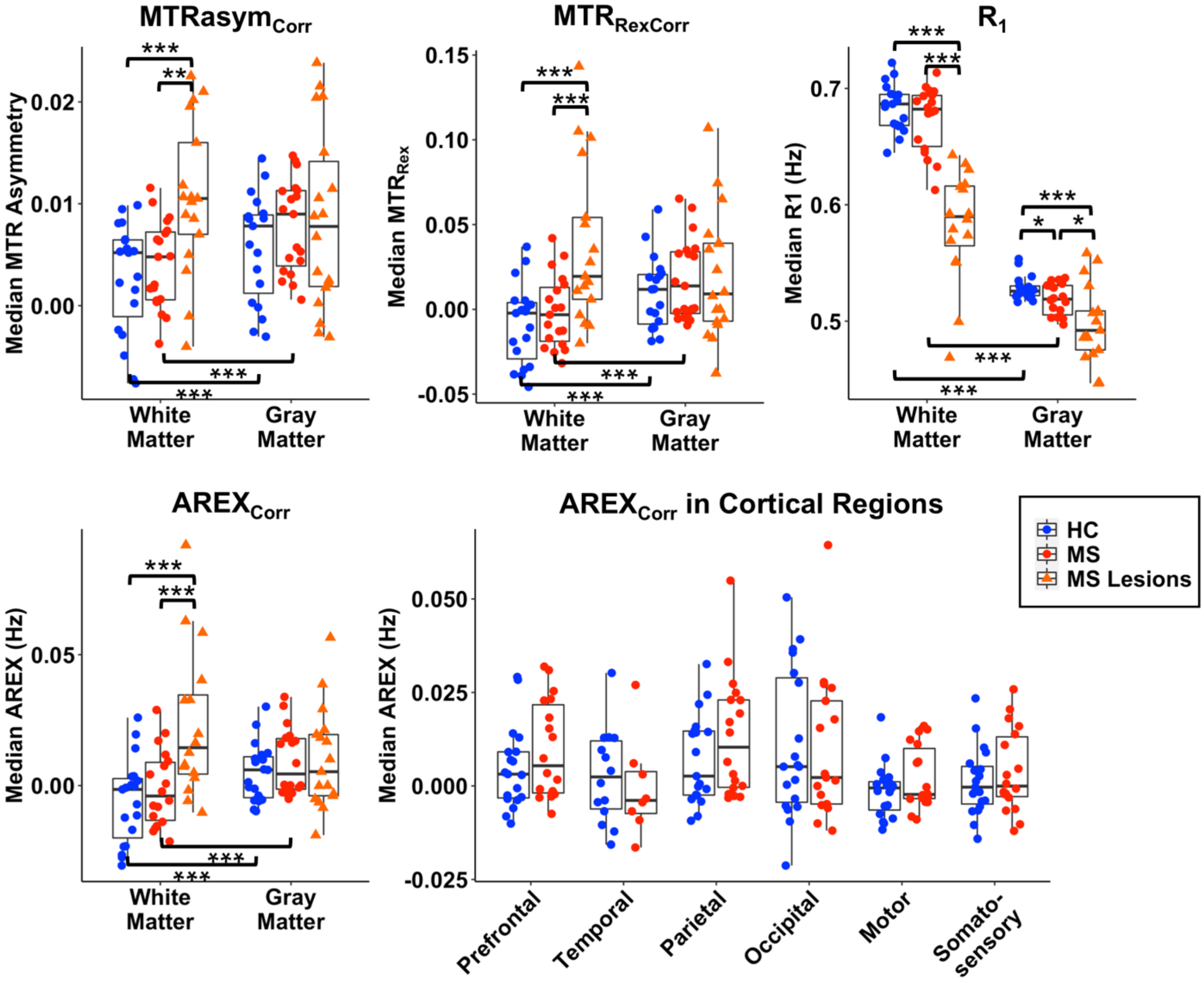

Supplementary Figure 1. Median values of MTRasym$_{Corr}$, MTR$_{RexCorr}$, AREX$_{Corr}$, and R_1 are shown for WM, GM, WM lesions, and cortical lesions for group comparisons. AREX$_{Corr}$ values are also shown for each cortical GM region. WM lesion values differ significantly from normal-appearing WM in MS patients and from healthy control (HC) WM for all CEST-derived indices and for R_1. Significant differences in GM and cortical lesions are only present in R_1. No group differences are significant for AREX$_{Corr}$ within cortical regions. All indices differ significantly between GM and WM within each group. *$p<0.05$, **$p<0.01$, ***$p<0.001$

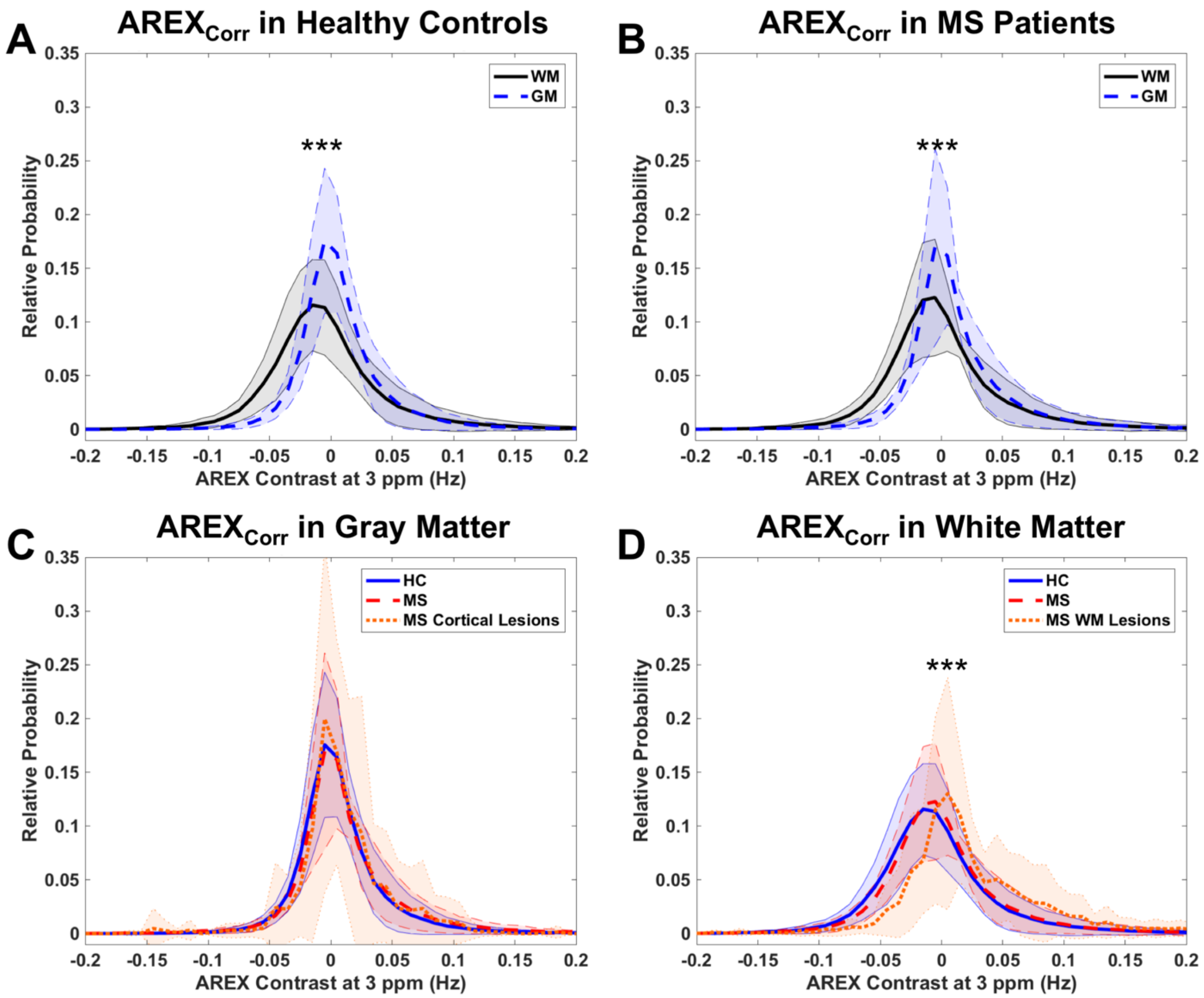

Supplementary Figure 2. Average histograms of AREX$_{Corr}$ values are shown for WM, GM, WM lesions, and cortical lesions for within-group **(A, B)** and between-group **(C, D)** comparisons (shaded area represents standard deviation). AREX$_{Corr}$ values are significantly greater in GM than in WM within the healthy control (HC) and MS patient groups. AREX$_{Corr}$ is increased significantly in WM lesions compared to normal-appearing WM in MS patients and compared to WM in healthy volunteers. ***$p<0.001$ for mean value comparison.

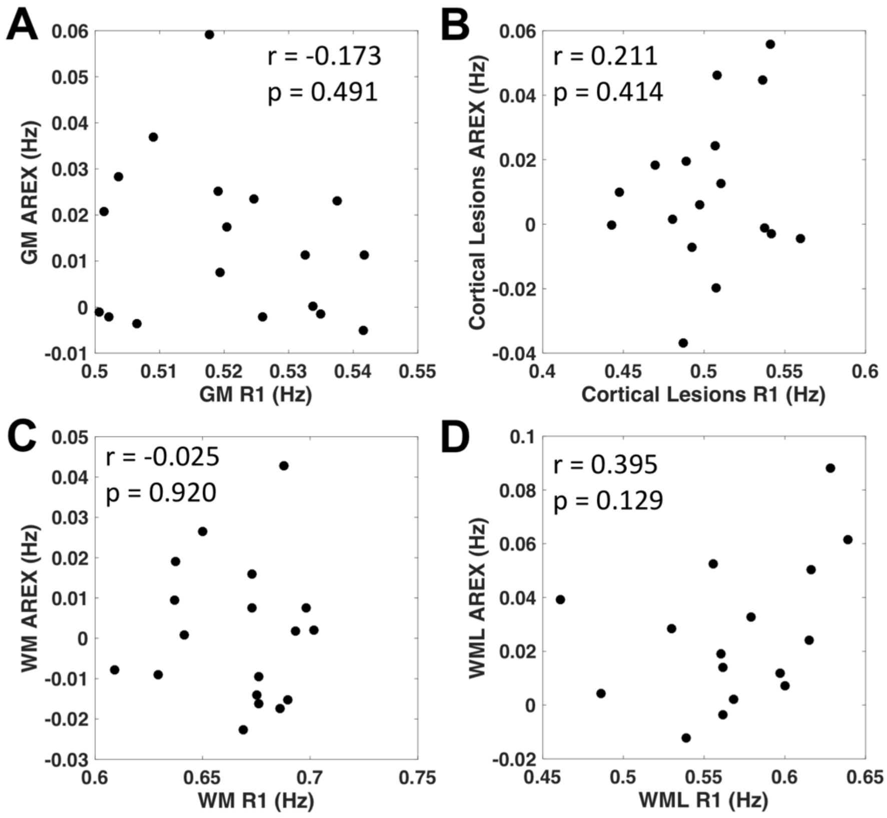

Supplementary Figure 3. Mean $AREX_{Corr}$ values do not correlate with corresponding mean R_1 values in each tissue region of interest in patients with MS. Plots are shown for normal-appearing GM **(A)**, cortical lesions **(B)**, normal-appearing WM **(C)**, and WM lesions **(D)** with the corresponding Pearson correlation coefficient r and p-value.
